# Viral Abundance and Diversity of Production Fluids in Oil Reservoirs

**DOI:** 10.3390/microorganisms8091429

**Published:** 2020-09-17

**Authors:** Liangcan Zheng, Xiaolong Liang, Rongjiu Shi, Ping Li, Jinyi Zhao, Guoqiao Li, Shuang Wang, Siqin Han, Mark Radosevich, Ying Zhang

**Affiliations:** 1 Key Laboratory of Pollution Ecology and Environmental Engineering, Institute of Applied Ecology, Chinese Academy of Sciences, Shenyang 110016, China; zhengliangcan18@mails.ucas.ac.cn (L.Z.); shirongjiu@iae.ac.cn (R.S.); liping315@mails.ucas.ac.cn (P.L.); wangshuangsunny@foxmail.com (S.W.); hansq@iae.ac.cn (S.H.); 2 University of Chinese Academy of Sciences, Beijing 100049, China; 3 Department of Biosystems Engineering and Soil Science, The University of Tennessee, Knoxville, TN 37996, USA; xliang5@vols.utk.edu (X.L.); mradosev@utk.edu (M.R.); 4 No. 2 Oil Production Company, Daqing Oilfield Limited Company, Daqing 163414, China; 18904893123@189.cn (J.Z.); liguoqiao_2001@163.com (G.L.)

**Keywords:** production fluids, oil reservoir, viral abundance, viral diversity, VBR

## Abstract

Viruses are widely distributed in various ecosystems and have important impacts on microbial evolution, community structure and function and nutrient cycling in the environment. Viral abundance, diversity and distribution are important for a better understanding of ecosystem functioning and have often been investigated in marine, soil, and other environments. Though microbes have proven useful in oil recovery under extreme conditions, little is known about virus community dynamics in such systems. In this study, injection water and production fluids were sampled in two blocks of the Daqing oilfield limited company where water flooding and microbial flooding were continuously used to improve oil recovery. Virus-like particles (VLPs) and bacteria in these samples were extracted and enumerated with epifluorescence microscopy, and viromes of these samples were also sequenced with Illumina Hiseq PE150. The results showed that a large number of viruses existed in the oil reservoir, and VLPs abundance of production wells was 3.9 ± 0.7 × 10^8^ mL^−1^ and virus to bacteria ratio (VBR) was 6.6 ± 1.1 during water flooding. Compared with water flooding, the production wells of microbial flooding had relative lower VLPs abundance (3.3 ± 0.3 × 10^8^ mL^−1^) but higher VBR (7.9 ± 2.2). Assembled viral contigs were mapped to an in-house virus reference data separate from the GenBank non-redundant nucleotide (NT) database, and the sequences annotated as virus accounted for 35.34 and 55.04% of total sequences in samples of water flooding and microbial flooding, respectively. In water flooding, 7 and 6 viral families were identified in the injection and production wells, respectively. In microbial flooding, 6 viral families were identified in the injection and production wells. The total number of identified viral species in the injection well was higher than that in the production wells for both water flooding and microbial flooding. The Shannon diversity index was higher in the production well of water flooding than in the production well of microbial flooding. These results show that viruses are very abundant and diverse in the oil reservoir’s ecosystem, and future efforts are needed to reveal the potential function of viral communities in this extreme environment.

## 1. Introduction

Diverse microbial communities inhabit oil reservoirs and have critical roles in mediating ecological processes. A wide range of microbes with various metabolic features can produce metabolites, such as surfactants, small organic acids, and biogas, that have oil displacement effects which is the main mechanism for enhancing oil recovery [1,2,3]. However, there is a lack of systemic understanding for the metabolic regulation of microbial communities in oil reservoir ecosystems, and microbial regulation remarkably impede the development and application of microbial enhanced oil recovery (MEOR) technology.

Viruses, the smallest and most numerous biotic agents [4], exist in various ecosystems and may be an important factor in controlling microbial populations and influencing microbial diversity [5,6]. Viruses could induce microbial cell lysis and release dissolved organic carbon and particulate organic carbon from inside host cells into the extracellular environment. These resources may be reused by other surrounding microbes further facilitating biogeochemical cycles in the biosphere [7,8]. In addition, many viruses are able to integrate their genome into their host chromosome and act as biological agents for horizontal gene transfer through lysogenic cycles contributing to microbial evolution. Although a lot of research has been conducted regarding viral ecology in soil [9,10,11,12,13,14,15], marine [4,8,16,17,18], river [19], wet-land [20] and paddy [21] environments, little effort has been made to study viral populations and viral communities in the oil reservoir. Microbial ecology in oil reservoirs mainly focuses on the relationship between injected and indigenous microbes, and the effects of injected nutrients and environmental factors on microbes and microbial community composition in the reservoir. It began in 1996, Voordouw [22] in the University of Calgary investigated the sulfur bacteria community in the oil fields of northern Canada through the most advanced molecular biological methods available at that time. The main purpose of studying the microbial ecology of oil reservoirs is to improve oil recovery rate, and the pilot—and full-scale of research efforts especially focus on the microbes, such as methanogenic archaea, fermentative bacteria, iron-reducing bacteria, and sulphate-reducing bacteria that have an important role in enhancing the oil recovery [23]. Scientists have shown that the microbial communities in oil reservoirs fluctuated during microbial enhanced oil recovery [24], and the reason for this phenomenon is still a mystery to date. Viruses are directly associated with host physiology and mortality and further influence microbial community dynamics in aquatic environments [18,25,26,27,28]. Therefore, viruses in the special environment of oil reservoir may be one of the major causes for the dynamic changes of the microbial community within the reservoir.

During an oilfield’s secondary exploitation, water-flooding is the most common oil recovery technique, in which water is injected to physically displace the remaining oil inside the reservoir and convey the replaced oil to the adjacent production well where petroleum can be collected [29]. Compared with water flooding, microbial flooding technology represents the use of microorganisms or their metabolites to extract the remaining oil from reservoirs. Microbial enhanced oil recovery is more feasible in oil reservoirs with temperatures lower than 80 °C and is very close to commercial application in China due to high success rate, low environmental impact, and cost-efficiency [30,31]. This study aimed to investigate viral abundance and diversity in the injection and production wells of water-flooding and microbial-flooding block in oil reservoirs. 

## 2. Materials and Methods

### 2.1. Site Description, Sample Collection and Pre-Treatment

The Daqing oilfield is located in the Songliao basin in northeastern China. The oilfield, discovered on 26 September 1959 and run by PetroChina, is the largest oilfield and oil production base in China. For this oilfield, the production depths ranged from 900 to 1200 m, the ambient temperature in the oil reservoirs ranges from 40 to 45 °C [32,33]. Most of the high permeability reservoirs have been explored by water flooding and polymer flooding [34]. In the meantime, there have been 12 field tests of microbial flooding recovery (MFR) conducted in Daqing oilfield [31].

**Figure 1:**
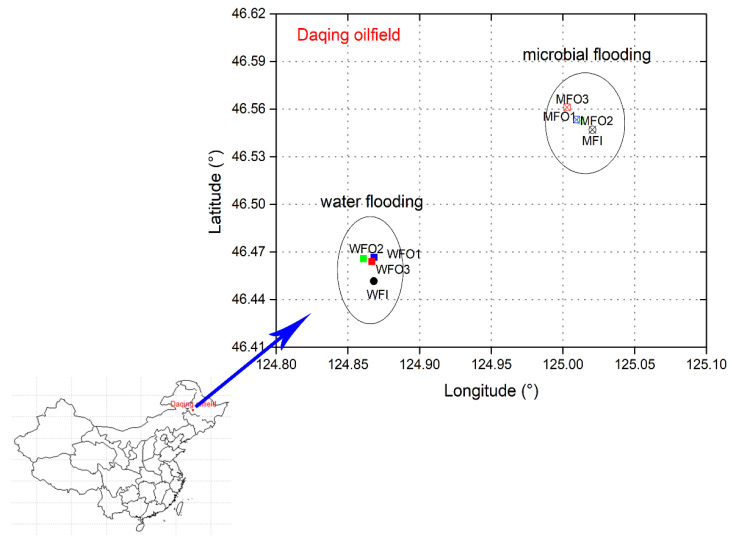
Sampling location for this study in the Daqing oilfield, China.

Samples (water and oil mixtures) were collected from two blocks developing long-term water-flooding and microbial-flooding experiments in Daqing oilfield, respectively, and nutrient or activator (single or complex inorganic compound) only were injected in microbial-flooding experiments. A total number of eight samples from eight sampling locations were collected in July, 2019, including one injection well (WFI) and three production wells (WFO1, WFO2, WFO3) in water flooding block and one injection well (MFI) and three production wells (MFO1, MFO2, MFO3) in microbial flooding block. (Figure 1). For each sample, 10 L oil and water mixture was collected from the sampling valve of the well and stored in a sterilized plastic bucket. The collected samples were stored on ice and immediately shipped to our laboratory (Shenyang, Liaoning, China) for further processing.

Upon arrival at the lab, each sample was fully mixed and filtered through filter paper (Φ 150 mm, pore size of 30–50 μm, Jinteng, Shanghai, China) to remove floating oil and large solid matter with the filtrate further filtered with a glass fiber filter membrane (Φ 50 mm, pore size of 0.45 µm; Jinteng). Forty mL of the filtrate was transferred into a sterilized 50 mL centrifuge tube, with formaldehyde (filtered with 0.22 µm filter membrane, Jinteng) immediately being added to a final concentration of 3.5% (*v*/*v*). Each solution was then rapidly frozen with liquid nitrogen and stored at −80 °C for further analysis of bacterial abundance. The remaining filtrate of each sample was filtered through 0.22 µm filter membranes (Φ 50 mm, pore size of 0.22 µm; Jinteng) for separation of bacteria and virus-like particles (VLPs). The filtrates (VLPs suspensions) were transferred into sterilized 50 mL centrifuge tubes and labelled. Sterilized formaldehyde was added to each VLPs suspension to a final concentration of 3.5% (*v*/*v*) which was then rapidly frozen with liquid nitrogen and stored at −80 °C for VLP enumeration. For virome analyses, 5 L of VLP suspension of each sample was transferred into a sterilized container, with addition of a final concentration of 0.1% FeCl_3_ (10 g/L). The mixtures were placed at ambient temperature (about 19–24 °C) for 8h to allow flocculation of VLPs and then centrifuged at 12,000 rpm/min for 30 min. The floccules were collected and flash frozen with liquid nitrogen and stored at −80 °C for future virome extraction.

### 2.2. Epifluorescence Microscopy

The extracted bacteria and VLPs were enumerated using epifluorescence microscopy according to the method described by Liang et al., [5] and Thurber et al., [35]. Briefly, triplicate subsamples of thawed bacteria and VLPs extracts were treated with Deoxyribonuclease (DNase) I enzyme (Takara, Kyoto, Japan) to digest free extracellular DNA. After DNase I treatment, subsamples of each VLP extract were vacuum filtered through a 0.02 µm pore-size Anodisc filter (Whatman, GE Healthcare Company, Maidstone, England) thereby capturing VLPs in the solution. Each bacterial solution was filtered through a 0.22 µm pore size filter (Jinteng) for separation of bacterial cells. Bacteria and viruses trapped on respective filters were stained with SYBR gold (Thermo Fisher Scientific, Waltham, MA, USA; at final concentration of 2 ×, 1:5000 dilution of original stock). The filters were counted immediately using epifluorescence microscopy (Olympus BX-53, Tokyo, Japan) with fluorescence (495 nm excitation wavelength and 537 nm emission wavelength). Images of bacteria and VLPs on the filters were taken using the camera and finally analyzed using Image J software [36].

### 2.3. Virome Extraction and Sequencing

Total viral nucleic acids were extracted from the Fe-virus floccules. The corresponding Fe-virus floccules of eight samples were sent to MAGIGENE Biotech Co., Ltd. (Guangzhou, China) (http://www.magigene.com/) for virome extraction and sequencing. For each sample, the Fe-virus floccules were resuspended using 10 mL of a resuspension buffer (0.25 mol/L ascorbic acid, 0.2 mol/L magnesium EDTA (Mg_2_EDTA), pH 6–7) at 4 °C [37]. DNase I was added to the suspension to remove free extracellular DNA. Amplification of bacterial 16S rRNA genes with primer 27F/1492R was performed to show the presence of bacterial DNA contamination. The total viral nucleic acids were extracted by using a MagPure Viral DNA/RNA Mini LQ Kit (R6662-02; Magen, Guangzhou, China) according to the manufacturer’s protocol. Extracted viral nucleic acids were amplified using a REPLI-g Cell WGA & WTA Kit (150054, Qiagen, Hilden, N.W, Germany) following the manufacturer’s protocol. Amplified viral nucleic acids were quality checked using a Qubit^®^ dsDNA HS Assay Kit (Life Technologies, Carlsbad, CA, USA) and then randomly sheared by ultrasound sonication (Covaris M220) to produce fragments of ≤ 800 bp, and sticky ends repaired and adapters added. Fragments of approximately 350 bp were collected by beads after electrophoresis using a D1000 screen Tape and D1000 Reagent (Agilent Technologies, Santa Clara, CA, USA). Libraries were finally pooled and subjected to 150 bp paired-end sequencing using the Novaseq 6000 platform (Illumina, San Diego, CA, USA).

### 2.4. Virome Data Analysis

Raw sequencing reads were processed to remove low-quality sequencing reads. Briefly, SOAPnuke (version 1.5.6) [38] was used to remove adapter sequences and reads (i) with more than 5% Ns; (ii) those with 20% base quality values less than 20; (iii) those arising from PCR duplications; as well as (iv) those with a polyA sequence (ATGC). The clean reads were *de novo* assembled using MEGAHIT version 1.1.2 with default parameters [39]. The assembled contigs were mapped against an in-house virus reference database separated from the GenBank non-redundant nucleotide (NT) database for identification of virus reads by BLASTx version 2.9.0+ [40] using alignment similarity ≥ 80%, length of matched area ≥ 500 bp and E-value ≤ 10^−5^. Contigs with significant hits were confirmed as virus sequences. Based on an in-house viral reference database separated from the GenBank non-redundant nucleotide (NT) database, viral contigs were assigned to taxa with Blast+ [41] using the default LCA algorithm parameters, and low-quality aligned reads with coverage less than 5 were filtered to improve the accuracy of taxonomic results. Viral diversity was evaluated by calculating the Shannon Diversity indices.

## 3. Results

### 3.1. VLP and Bacterial Abundance

**Figure 2:**
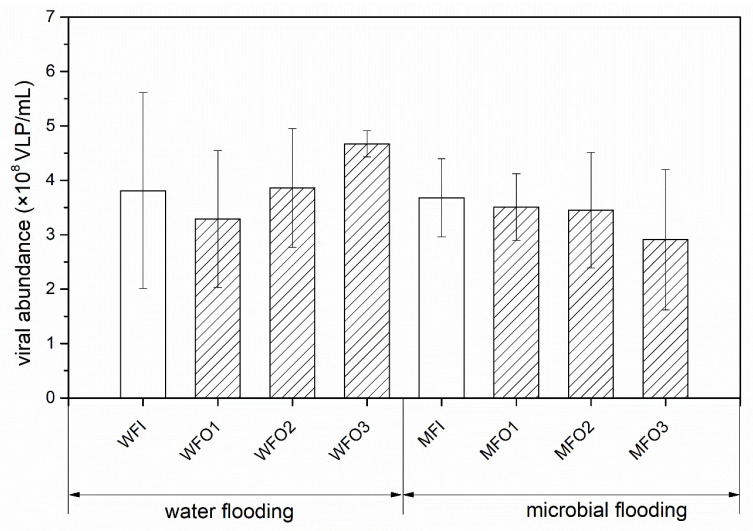
Viral abundances in production fluids from Daqing oilfield. The bars with slash represent production wells, and bars without slash represent the injection well.

The VLPs abundances in water flooding and microbial flooding block are shown in Figure 2. The results showed that the VLP abundance was 3.8 ± 2.6 × 10^8^ VLPs/mL in injection wells of the water flooding block and 3.9 ± 0.7 × 10^8^ VLPs/mL in production wells of the water flooding block. In the microbial flooding block, the VLP abundance in the injection wells was 3.7 ± 0.7 × 10^8^ VLPs/mL, which was higher than that in production wells (3.3 ± 0.3 × 10^8^ VLPs/mL). No significant difference (*p* > 0.05) in VLPs abundance was detected between the injection and production wells in both blocks of water flooding and microbial flooding.

**Figure 3:**
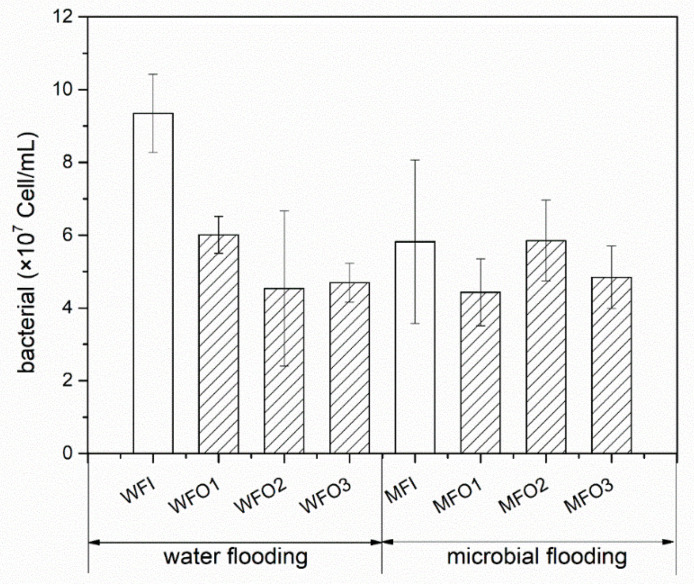
Bacterial abundances in production fluids from the Daqing oilfield. The bars with slash represent production wells, and bars without slash represent the injection well.

In the water flooding block, bacterial abundance (10^7^ cells/mL) in injection well was 9.3 ± 1.1 and was significantly higher than its population (5.1 ± 1.3) in production wells (*p* = 2 × 10^−4^) (Figure 3). However, in microbial flooding block, the density of bacteria (10^7^ cells/mL) in the injection wells was 5.8 ± 2.3, which was similar to that in the production wells (5.0 ± 1.1). There were no significant differences (*p* > 0.05) in bacterial abundance between the production wells in both water flooding and microbial flooding blocks.

### 3.2. Viruses to Bacteria Ratio

**Figure 4:**
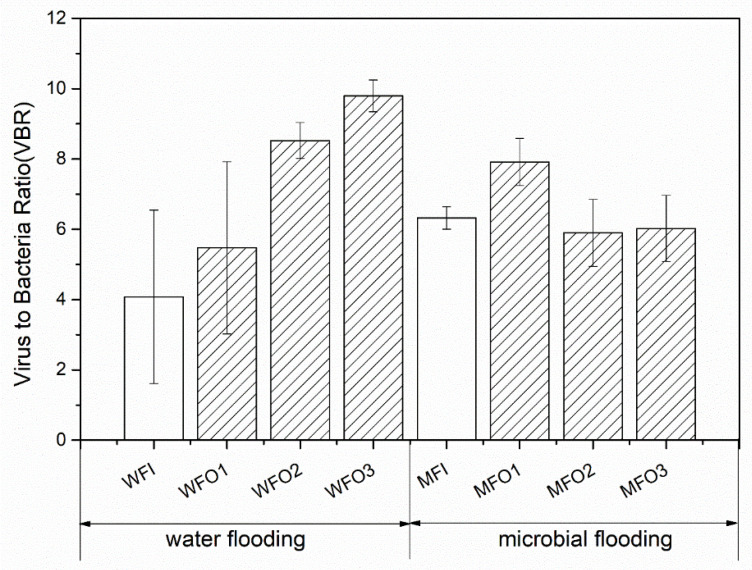
The ratio of viral to bacterial abundance for each sample in the Daqing oilfield. The bars with slash represent production wells, and bars without slash represent the injection well.

The ratio of VLPs to bacterial abundance (VBR, Figure 4) was calculated from the viral and bacterial abundance. The results suggested that, for water-flooding, The VBR in injection well was 4.1 whereas the VBR determined for production wells varied from 5.5 to 9.8, with a mean of 7.9 ± 2.2; Interestingly, the VBR for the injection wells were significantly different from the values determined for production wells (*t*-test, *p* = 0.003), and there were significant differences between WO1 and WFO3 (*t*-test, *p* = 0.006), however no significant differences between WFO2 and WFO3 (*t*-test, *p* = 0.993) or between WFO1 and WFO2 (*t*-test, *p* = 0.236); For microbial-flooding, VBR in the injection well was 6.3, and VBR in production wells ranged from 5.5 to 7.9, with a mean of 6.6 ± 1.1. Our results indicated that no statistically significant differences (*p* > 0.1) among 3 production wells, between water-flooding and microbial-flooding, and between the injection and production wells.

### 3.3. Viral Biodiversity

#### 3.3.1. Viral Taxonomic Classification

A total of 256,298,949 quality-controlled valid reads (150 bp in length) were obtained from 8 samples. Megahit (version 1.1.2) [39] was used to assemble the sequencing reads of samples into contigs thereby generating 253712, 98943, 44000, 132111, 166617, 41931 and 170199 contigs in WFI, WFO1, WFO2, WFO3, MFI, MFO1, MFO2, and MF03, respectively (Table 1). GC content variation among contigs ranged from 40 to 48%. These contigs would be further taxonomically annotated. All the contigs were mapped using blastx version 2.9.0+ [40] with an E-value threshold of lower than 10^−5^ to an in-house viral reference database that was extracted from NT database in NCBI database. For water flooding block, 36.64% of contigs in injection wells were annotated as virus, while the percentage identified as virus in production wells was 35.34%. In the microbial flooding block, the percentage of the contigs annotated as viral was 26.36 and 55.94% in the injection and production wells, respectively. The proportion of phages in virus category was similar among all samples, except that the percentage in water flooding samples (25.75 ± 2.15%) was slightly lower than in microbial flooding samples (28.57 ± 1.60%) (Table 2).

#### 3.3.2. Viral Community Structure

**Figure 5:**
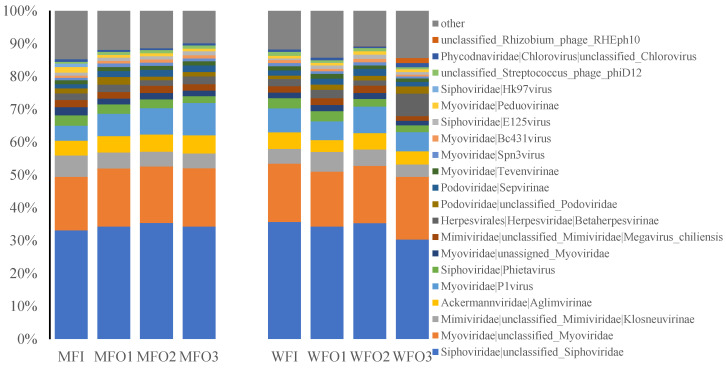
The relative abundance of the dominant viral families in each well.

The viral families with a relative abundance higher than 1% were determined as dominant families (Figure 5). *Myoviridae*, *Siphoviridae*, *Podoviridae*, *Herpesvirales*, *Ackermannviridae*, *Mimivirdae* and *Phycodnaviridae* were the most common and dominant viral families across all samples, and these viral families together accounted for 82–88% of the total sequences of each sample. It was notable that the relative abundance of tailed phages, including *Myoviridae*, *Siphoviridae*, and *Podoviridae*, ranged from 65 to 74% in all samples. *Siphoviridae*, *Myoviridae*, *Mimiviridae*, *Ackermannviridae*, *Podoviridae*, and *Herpesvirales* had a relative abundance higher than 1% in both the injection well and production wells of the water flooding block, however, in injection well of water flooding block, one viral family (*unclassified streptococcus phage_phiD12*) was specific family. And for microbial flooding block, six dominant viral families (*Siphoviridae*, *Myoviridae*, *Mimiviridae*, *Ackermannviridae*, *Podoviridae*, and *Herpesvirales*) were both identified in injection and production wells. but one unclassified viral family (*unclassified streptococcus phage_phiD12*) was only observed in injection well of water flooding block; the number of dominant viral families in injection well was the same as in production wells for microbial flooding block; therefore, we could know that annotated dominant viral families in water flooding block were more than in microbial flooding block.

**Figure 6:**
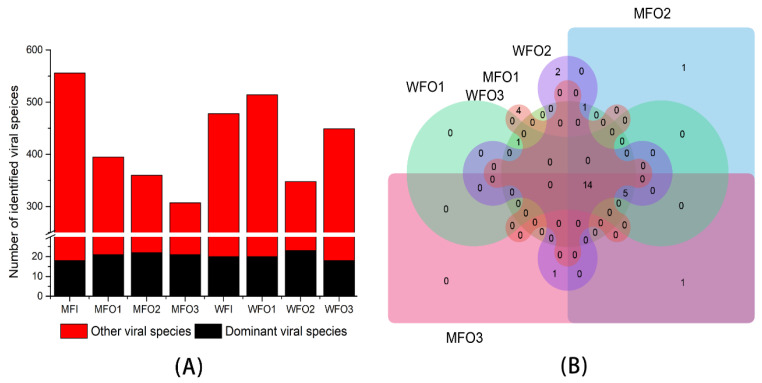
(**A**) The viral species richness in each production water sample, boxes with red represent dominant viral species of each sample that each viral relative abundance was more than 1%, and boxes with black represent the remaining viral species. (**B**) The number of shared or specific dominant viral species in production wells of water flooding and microbial flooding blocks.

After contigs of all samples were annotated into viral taxonomy, for each sample, we selected the species that the relative abundance was higher than 1% as dominant viral species at the species level in this study. A total of 478, 541, 348, 449, 556, 395, 360 and 307 viral species were identified, of which 20, 20, 23, 18, 18, 21, 22 and 21 viral species were dominant species in WFI, WFO1, WFO2, WFO3, MFI, MFO1, MFO2 and MFO3, respectively (Figure 6A). There were 14 and 19 common dominant viral species in three production wells of water flooding block (WFO1~3) and 3 production wells of microbial flooding block (MFO1~3), respectively. Among production wells of water flooding and microbial flooding block, 14 dominant viral species were common (Figure 6B).

**Figure 7:**
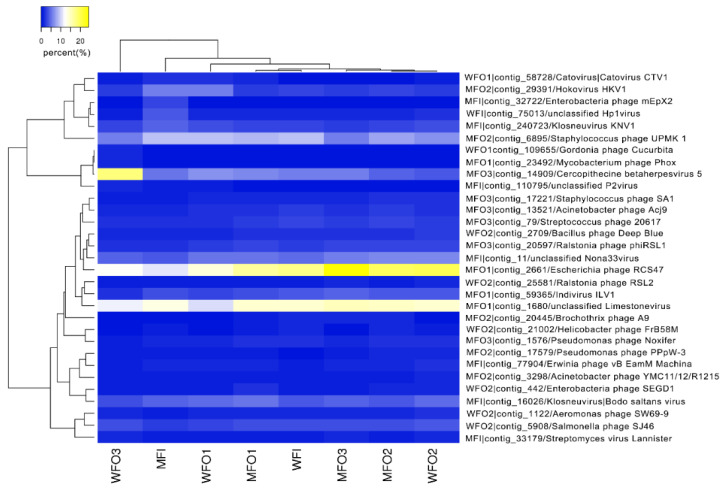
The heatmap of annotated dominant viral species in each well.

The heatmap showed that (Figure 7), the relative abundance of the dominant viral species such as *Escherichia phage RCS47*, *unclassified Limestonevirus*, *Staphylococcus phage UPMK 1*, *Cercopithecine betaherpesvirus 5* and *Bodo saltans virus* were different between injection and production wells and among 3 production wells in water flooding. The dominant viral species’ relative abundances in wells of microbial flooding were similar to those in the water flooding wells. The Virus-Host database (Virus-Host Database, https://www.genome.jp/virushostdb/) was used for revealing the host of the dominant viral species in all wells, and the results showed that 45.16% of the dominant viral species were paired to corresponding hosts (Table 3).

#### 3.3.3. Viral Diversity Analysis

**Figure 8:**
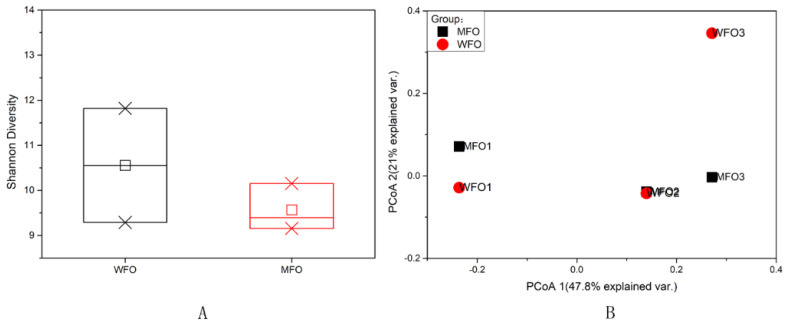
Viral community diversity analysis. (**A**) Shannon diversity index of viral communities. (**B**) PCoA of viral communities in the production wells of the water and microbial flooding block.

The viral diversity in production wells of the water flooding block (WFO) and the microbial flooding block (MFO) was assessed with Shannon diversity and Principal component analysis (PCoA). The mean Shannon index of viral communities in water flooding (10.55) was higher than microbial flooding (9.57); however, the difference was not significant (*p* > 0.05) between them (Figure 8A). The PCoA based on Bray-Curtis dissimilarity of the identified viral components showed that the first and second PCoA axes respectively explained 47.8 and 21% of the variance. The MFO samples were more closely clustered with each other than the WFO samples (Figure 8B).

## 4. Discussion

### 4.1. Viral and Bacterial Abundance in Oil Reservoir

This work explored the abundance of viruses and bacteria and showed that both viruses and bacteria were highly abundant in the oil reservoir ecosystem. As still little is known about viral community dynamics in petroleum reservoirs, more efforts are needed to investigate the viral distribution, abundance, and diversity in the underground ecosystem. Compared with the viral abundance in other environments, for example, aquatic environment, the abundance of viruses in the petroleum reservoir was in a different order of magnitude as those previously reported in aquatic environments, including marine [42,43,44], stormwater retention ponds [45], and tropical estuaries [46]. Interestingly, the virus abundance of production wells in the microbial flooding block was slightly higher than that in the water flooding block. Microbial activators (nutrients and/or inorganic compounds) were injected in injection wells of the microbial flooding block, which could activate in-situ microbial populations and furtherly provide more hosts for viruses, and the viral abundance in production wells was theoretically higher than those in water flooding block. However, all samples were collected from the production wells of microbial flooding block more than 30 d after injection and the abundance of viruses might return to the steady state before microbial injection occurred, which would account for the similarity in viral abundance observed in the production wells of the water—and microbial flooded wells.

The abundance of bacteria in the production water of the oil reservoir was approximately 5 × 10^7^ cell per milliliter, which was two orders of magnitude higher than previously published [47] and was at a similar level to the total cell density determined by means of a counting chamber [48]. When using EFM with SYBR Gold dye to count bacteria, the dye combines with the DNA in the sample to produce fluorescence signals, which not only count culturable bacteria, but also unculturable bacteria, and bacterial numbers were counted in oil reservoir by using colony forming unit (CFU) method on the plate by most probable number (MPN) in most of the published papers [15,47,48,49,50,51], which only enumerates culturable bacteria, and bacterial abundance may be significantly underestimated in the sample itself.

The ratio of VLPs to bacterial abundance (VBR) has been used to study the relationship between viruses and bacteria in the environment [52]. VBR can be influenced by a comprehensive balance of factors, such as the viral production, the transport of viruses through sinking particles, decay rates and life strategies [16]. Normally high VBR values are attributed to high ongoing viral infections, while low VBR values may often be interpreted as diminished viral activity or high viral decay rates. Parikka and his coworkers [53] analyzed virus-to-prokaryote ratio in a variety of ecosystems including samples from coastal, estuary, open ocean, river, lake, and pond environments, and their results showed that mean VPR values varied from 5.6 to 28.5. To the best of knowledge, there was no existing reports of viral abundance in the oil reservoir’s environment, and here we reported high viral abundances and varying VBRs in the studied oil reservoirs shed light on the relationship between viruses and bacteria in this environment. The value of VBR in this work ranged from 4.1 to 9.8, which was also within this range reported by Parikka et al. [53].

### 4.2. Viral Community Diversity

In order to explore the viral diversity and viral community structure in oil reservoir, we conducted viral metagenomic sequencing. In this study, the contigs identified as virus ranged from 21.93 to 75.37%, but the majority (24.63 to 78.07%) of contigs still could not be identified by comparison to the known virosphere (based on the NT database). This phenomenon has been reported previously for viromes in other environments, including soil [5,10], marine [17], freshwater [54], etc. These contigs that originate from viruses but do not align to any reference virus sequence have been referred to as “viral dark matter” [55]. Only 19.24–29.91% of contigs showed the similarity to known phages, which was far lower than marine [17], tropical freshwater [54] and soil [5,10]. In contigs identified as viruses, dominant viral families with relative abundance more than 1% included *Myoviridae*, *Siphoviridae*, *Podoviridae*, *Herpesvirales*, *Ackermannviridae*, *Mimivirdae* and *Phycodnaviridae*, which mainly belonged to tailed phages (*Myoviridae*, *Siphoviridae*, and *Podoviridae*,). Many viral sequences were also closely related to *Caudovirales* in other environments [13,14,56,57]. However, the corresponding proportion of the dominant families were completely different in the water-flooding and microbial flooding blocks, which could be explained by that type of ecosystem mainly drove the viral community structure [12] and special environmental conditions of oil reservoirs.

*Acinetobacter* phage *phiAC-1* was a dominant viral species only observed in production wells of the microbial flooding block but not in the water-flooding block, and its host (*Acinetobacter soli*) was found in the Virus-Host Database. In the microbial flooding block, microbes were injected in the injection well, which strengthened the in-site microbial activities and further producing biofilms benefiting most microbes through support and protection to the microbes from physical and chemical stresses, and mediating mineral dissolution by *Acinetobacter soli* [58,59]. These microbial-mediated processes might be able to activate *Acinetobacter soli* activities and further enhance *Acinetobacter phage phiAC-1* production as a results of *Acinetobacter phage phiAC-1* infecting *Acinetobacter soli*. The analysis of viral community diversity in production wells of water-flooding and microbial flooding block showed that the diversity of the viral communities in the microbial flooding block was lower than that in the water-flooding block, which could be attributed to the cooccurring microbial biodiversity in the environment. Neu et al., [60] showed that the bacterial component of the microbiota can directly or indirectly impact the outcome of infection of a range of different viruses. The work of Liang et al., [9] also suggested that the lysogenic reproductive strategies of viral communities were correlated with bacterial diversity. In the investigations of the microbial biodiversity in oil reservoir, Sun et al. [61] reported that the relationship between the bacterial biodiversity was positively related with water content in MEOR experiments, and Li et al., [33] also reported that the Shannon index of bacterial communities in production water of water flooding was higher than those in polymer flooding in the Daqing oilfield in China. In our study, the viral communities in the production wells of microbial flooding were significantly different from those in the production wells of water flooding, which might be caused by not only microbes injection, but also nutrients [62], depths [9], environment variation and spatial distance [63].

## 5. Conclusions

This study shows that viruses and bacteria were extremely abundant both in the injection and production wells during water and microbial flooding. Interestingly, the results suggest that the method used for enhancing oil recovery had no significant effects on VLP abundance in the oil wells, VLP abundance in the injection wells was not significantly different from those in production wells either. However, for water flooding block, the bacterial abundance in the production wells was significantly higher than the injection wells, and VBR was lower in the production wells than in the injection well. More than three hundred viral species were detected in all samples, and most contigs identified as virus belonged to tailed phages. The composition of dominant viral species was similar across the samples, but the relative abundance of each species was highly different in each sample. The results showed that viruses are abundant in production water of oil reservoir and might be prevalent in the oilfield ecosystem which suggests potential function of viruses in microbial processes. Viral ecological data are helpful for the development of understanding microbial ecosystem functioning in the oil reservoir environment. This work as the first study of viral ecology in oil reservoir extends the scope of research in the field of environmental virology and provides a foundation for future work in oilfield microbial ecology and screening phages for functional microorganisms in oilfields.

## Figures and Tables

**Table 1:** The result of virome sequences from oil reservoir production water samples.

Sample ID	Number of Reads (PE)	Total Number of Bases (Mb)	Total Number of Contig	GC Content (%)	Longest Contig Size (bp)	Shortest Contig Size (bp)	% Total Abundance	
							Annotated as Virus	Unclassified
WFI	34089873	311.56	253712	41.68	232481	300	36.64	63.36
WFO1	30623735	116.67	98943	47.25	567560	300	27.72	72.28
WFO2	24602901	46.28	44000	39.73	96276	300	56.37	43.63
WFO3	24137426	32.55	26033	45.87	234881	300	21.93	78.07
MFI	28158702	156.98	132111	40.45	290374	300	26.36	73.634
MFO1	42093578	198.6	166617	45.31	316529	300	31.56	68.44
MFO2	26647904	41.41	41931	41.35	62494	300	60.90	39.10
MFO3	45944830	203.86	170199	48.38	425897	300	75.37	24.63

**Table 2:** Percentage of viral contigs, non-viral contigs, phage contigs resulting from sequencing reads of viromes from samples.

Sample	Percentage of Annotated Contigs (%)	Viral Percentage in Annotated Contigs (%)	Non-virus Percentage in Annotated Contigs (%)	Phage Percentage in Annotated Viral Contigs (%)
WFI	50.78	36.64	63.36	21.64
WFO1	37.3	27.72	72.28	23.33
WFO2	72.01	56.37	43.63	26.45
WFO3	27.78	21.93	78.07	27.46
MFI	38.05	26.36	73.64	19.24
MFO1	38.36	31.56	68.44	29.91
MFO2	77.98	60.90	39.1	26.79
MFO3	92.27	75.37	24.63	29.00

**Table 3:** The annotated dominant viral species and corresponding host for all wells.

Virus	Host
*Escherichia phage RCS47*	*Escherichia coli*
*Cercopithecine betaherpesvirus 5*	*Cercopithecus*
*Salmonella phage SJ46*	*Salmonella enterica subsp. enterica serovar Indiana*
*Ralstonia phage phiRSL1*	*Ralstonia solanacearum*
*Streptococcus phage 20617*	*Streptococcus thermophilus DSM 20617*
*Acinetobacter phage Acj9*	*Acinetobacter johnsonii*
*Pseudomonas phage Noxifer*	*Pseudomonas fluorescens SBW25*
*Bacillus phage Deep Blue*	*Bacillus cereus BtB2-4*
*Staphylococcus phage SA1*	*Staphylococcus aureus*
*Erwinia phage vB EamM Machina*	*Erwinia amylovora*
*Brochothrix phage A9*	*Brochothrix thermosphacta*
*Ralstonia phage RSL2*	*Ralstonia*
*Pseudomonas phage PPpW-3*	*Pseudomonas plecoglossicida*
*Gordonia phage Cucurbita*	*Gordonia terrae*
